# A Complex Endomembrane System in the Archaeon *Ignicoccus hospitalis* Tapped by *Nanoarchaeum equitans*

**DOI:** 10.3389/fmicb.2017.01072

**Published:** 2017-06-13

**Authors:** Thomas Heimerl, Jennifer Flechsler, Carolin Pickl, Veronika Heinz, Benjamin Salecker, Josef Zweck, Gerhard Wanner, Stefan Geimer, Rachel Y. Samson, Stephen D. Bell, Harald Huber, Reinhard Wirth, Louie Wurch, Mircea Podar, Reinhard Rachel

**Affiliations:** ^1^LOEWE Research Center for Synthetic Microbiology (SYNMIKRO), Philipps University of MarburgMarburg, Germany; ^2^Plant Development and Electron Microscopy, Biocenter LMUMunich, Germany; ^3^Center for Electron Microscopy, University of RegensburgRegensburg, Germany; ^4^Institute of Experimental and Applied Physics, University of RegensburgRegensburg, Germany; ^5^Cell Biology and Electron Microscopy, University of BayreuthBayreuth, Germany; ^6^Molecular and Cellular Biochemistry Department, Indiana UniversityBloomington, IN, United States; ^7^Microbiology Department, University of RegensburgRegensburg, Germany; ^8^Biosciences Division, Oak Ridge National LaboratoryOak Ridge, TN, United States; ^9^Microbiology Department, University of TennesseeKnoxville, TN, United States

**Keywords:** Archaea, ultrastructure, 3D, eukaryogenesis, FIB/SEM, electron tomography, symbiosis, membranes

## Abstract

Based on serial sectioning, focused ion beam scanning electron microscopy (FIB/SEM), and electron tomography, we depict in detail the highly unusual anatomy of the marine hyperthermophilic crenarchaeon, *Ignicoccus hospitalis*. Our data support a complex and dynamic endomembrane system consisting of cytoplasmic protrusions, and with secretory function. Moreover, we reveal that the cytoplasm of the putative archaeal ectoparasite *Nanoarchaeum equitans* can get in direct contact with this endomembrane system, complementing and explaining recent proteomic, transcriptomic and metabolomic data on this inter-archaeal relationship. In addition, we identified a matrix of filamentous structures and/or tethers in the voluminous inter-membrane compartment (IMC) of *I. hospitalis*, which might be responsible for membrane dynamics. Overall, this unusual cellular compartmentalization, ultrastructure and dynamics in an archaeon that belongs to the recently proposed TACK superphylum prompts speculation that the eukaryotic endomembrane system might originate from Archaea.

## Introduction

The genus *Ignicoccus* belongs to the Desulfurococcaceae within the Crenarchaeota. Currently the genus comprises three species—*I. hospitalis* (Paper et al., 2007), *I. islandicus*, and *I. pacificus* (Huber et al., 2000). Additionally, a strain was isolated from the Alvin diving expedition to the East Pacific Ridge in 2008, MEX13A. They were all isolated from submarine hydrothermal vent systems or black smokers and are anaerobic, hyperthermophilic, obligate chemolithoautotrophs with an optimal growth temperature of 90°C. As an obligate energy source, *Ignicoccus* cells reduce elemental sulfur using molecular hydrogen as an electron donor. Carbon fixation is thought to occur via the dicarboxylate/4-hydroxybutyrate pathway (Jahn et al., 2007; Huber et al., 2008).

A taxonomic characteristic that distinguishes *Ignicoccus* cells from all other known Archaea is their unusual cell anatomy (for a review on archaeal cell structures see Klingl et al., 2013). Unlike most other Archaea, *Ignicoccus* cells lack a proteinaceous cell envelope (S-layer) and are instead surrounded by a double membrane system: an outer cellular membrane (OCM) and an inner (“cytoplasmic”) membrane (IM) (Näther and Rachel, 2004; Huber et al., 2012). Moreover, both membranes differ in their composition of lipids. The OCM consists solely of archaeol, whereas in the IM, caldarchaeol is also present, thus resulting in a partial monolayer (Jahn et al., 2004). In *I. hospitalis*, the most abundant protein in the OCM is Ihomp1, which is a 6.23 kDa protein that forms oligomeric pore complexes and has no recognizable homologs in other archaea (Burghardt et al., 2007, 2008). Both the H_2_:sulfur oxidoreductase and the archaeal A_1_A_*O*_ ATP synthase complexes are located in the OCM (Küper et al., 2010), which highlight a unique, remarkable functional compartmentalization of *Ignicoccus* cells: While replication, transcription, and translational processes occur in the cytoplasm, at least a few energy consuming processes have to take place in the so called inter-membrane compartment (IMC), which is encased by the IM and the OCM. Indeed, an (ATP-consuming) acetyl-CoA synthetase is located in the IMC associated with the OCM (Mayer et al., 2012).

The best-known and most studied *Ignicoccus* species is *I. hospitalis* since it enables the growth of *Nanoarchaeum equitans* on its cell surface. This system is especially appealing for investigating inter-species relationships since genomes of both organisms are very small (1.3 and 0.49 Mbp, respectively) (Waters et al., 2003; Podar et al., 2008a). While *I. hospitalis* can grow in pure culture, *N. equitans* only thrives in co-culture with *I. hospitalis*, at least under laboratory conditions (Huber et al., 2002). The nature of and mechanisms that enable this intimate association are still unclear (Jahn et al., 2008), though up to now, no beneficial effect for *I. hospitalis* has been found.

Our study addresses the 3D ultrastructure of *I. hospitalis* cells, with a special focus on the complex membrane system, and the interaction site with cells of *N. equitans*. Based on the results we were able to assign putative functions to several structures.

## Materials and methods

### Cell growth

The type strains KIN4/I, KIN4/M, LPC33, Kol8 as well as strain MEX13A were obtained from the Culture Collection of the Institute for Microbiology and Archaea Centre, University of Regensburg. Cells were grown in ½ SME medium at 90°C, as previously described (Huber et al., 2000; Paper et al., 2007) with elemental sulfur as the electron donor and a gas phase consisting of H_2_/CO_2_ (250 kPa; 80/20, v/v).

### Phase contrast microscopy

Phase contrast microscopy at 90°C and image acquisition was done as previously described (Horn et al., 1999).

### Sample preparation

In order to avoid centrifugation for concentrating cells and consequently providing the best possible preservation, we either grew *Ignicoccus* cells in cellulose capillaries (Rieger et al., 1997) or filtered them through a PVDF membrane with a pore size of 0.45 μm or a polycarbonate membrane with a 0.2 μm pore size. Samples were then high pressure frozen (EM PACT2, Leica, Wetzlar, Germany) and freeze-substituted (EM AFS 2, Leica, Wetzlar, Germany). For early preparations from which the 3D models based on serial sections originate, a variety of different freeze-substitution media were used (see Rachel et al., 2010). Later preparations for FIB/SEM, electron tomography, and immunolabeling were exclusively done with acetone containing 0.5% (v/v) glutardialdehyde, 0.5% (w/v) uranyl acetate, and 5% (v/v) water. All samples were embedded in Epon 812 substitute resin (Fluka Chemie AG, Buchs, Switzerland). Single and serial sections of 50 or 200 nm thickness were cut with a diamond knife (Diatome, Biel, Switzerland) mounted on a UC6 ultramicrotome (Leica, Wetzlar, Germany). More details on the protocol can be found in Rachel et al. (2010). For FIB/SEM analysis, the top of the Epon blocks was cut off with a saw and mounted on a SEM stub with conducting silver. Additionally, the samples were made conductive by evaporation of ~10 nm carbon on top.

### Electron microscopy and image acquisition

Micrographs and tilt series were taken on three TEMs: a 120 kV Philips CM12 (FEI, Hillsboro, OR, USA) equipped with a slow-scan CCD camera (TVIPS0124, 1k × 1k pixels; TVIPS GmbH, Gauting, Germany), a 200 kV JEOL JEM 2100 (Tokyo, Japan) with a 4k × 4k camera (UltraScan 4000; Gatan Inc., Pleasanton, CA, USA) and a JEOL JEM 2100F (Tokyo, Japan) with a 4k × 4k CMOS camera (TemCam-F416; TVIPS, Gauting, Germany). For imaging and tilt series acquisition, the software packages EM-Menu (TVIPS, Gauting, Germany) and SerialEM (Mastronarde, 2005) were used.

### Energy dispersive X-ray analysis

For EDX analysis, a Philips CM30 TEM (FEI, Hillsboro, OR, USA) equipped with a XFlash 5030 detector (Bruker AXS, Billerica, MA, USA) was used, together with the software Esprit/Quantax (Bruker AXS, Billerica, MA, USA). Sample analysis was done on 200 nm sections, covered by a thin layer of carbon (~10 nm).

### Focused ion beam/scanning electron microscopy

Acquisition of image stacks via FIB/SEM was performed on a Zeiss Auriga 60 (Zeiss, Oberkochen, Germany) in “slice and view” mode. 30 kV and 1 nA or 500 pA, respectively, were used for the gallium ion beam. For image acquisition, 1.5 kV, high current mode and an aperture of 60 μm were applied. In addition, an in-lens EsB detector with −1,400 V EsB-grid voltage was used.

### Image editing and 3D reconstruction

Basic image editing processes (cropping, binning, brightness, contrast, adjusting to 8bit scale, format converting, etc.), as well as noise reduction filtering (when necessary) were done using ImageJ (Abramoff et al., 2004). Stitching was done either in the respective image acquisition software or with a stitching plugin for ImageJ (Preibsch et al., 2009). Alignment of images of 50 nm section series was done using the software AMIRA (FEI, Hillsboro, OR, USA). For FIB/SEM data, the ImageJ plugin “StackReg” (Thévenaz et al., 1998) was used for alignment. For visualization, either the volume texture rendering tool “voltex” in AMIRA was used, or segmentation had been done manually (i.e., structures of interest were out-lined and color-coded for each single image of a series). A smoothing filter for the surface, as well as an interpolation process between the original slices, was applied in order to improve the virtual resolution of the 3D models in the z-axis. An interpolation was omitted for FIB/SEM and electron tomography data. For recording tilt series, 15 nm colloidal gold particles were applied onto the 200 nm sections as fiducial markers, and specimens were tilted to about ±65°. The reconstruction of the tomograms was done by the “simultaneous iterative reconstruction technique” (SIRT) algorithm of IMOD (Kremer et al., 1996). Consecutive tomograms were joined using IMOD. After obtaining the final tomograms, volumes were visualized in the same manner like serial sections and FIB/SEM data. Movies were created using AMIRA and ImageJ.

### Antibody generation and immunolabeling

Antibodies against *Ignicoccus* ATP-Synthase components were obtained as previously described (Küper et al., 2010). The gene encoding the V4R protein Igni_1332 was codon optimized, synthesized and cloned into the *E. coli* expression plasmid pJExpress by DNA2.0 (Menlo Park, CA, USA). The protein was purified on Ni resin based on a C-terminal 6xHis tag and used to raise an antibody in rabbits by Covance Research Products Inc. (Denver, PA, USA). Antibodies against Igni_0994 and Igni_0475 were obtained, starting from purification of genomic DNA using previously described methods (Ramakrishnan and Adams, 1995). The Igni_0475 open reading frame was amplified by PCR, using the primer pairs Ig475For (5′ GAATCC**CCATGG**TTAGCGGTAGAGCAGTC 3′) and Ig475Rev (5′ GGAATTC**GTCGAC**CTTCTTCCTCTCGTCCAAGA 3′). The PCR product was digested with NcoI and SalI (sites bolded and underlined in oligonucleotide sequences) and ligated into NcoI/XhoI digested pET33b. The resultant plasmid encoded Igni_0475 with a C-terminal hexa-histidie tag. Similarly, the Igni_0994 (Vps4) open reading frame was amplified with the primer pair IgVPS4For (5′ GAATTC**CATATG**AGTAGGCTGGACAGGTT 3′) and IgVPS4rev (5′ GGAATTC**GTCGAC**GAGCGCGCCGTGGGCCTTG 3′). The PCR product was digested with NdeI and SalI (sites bolded and underlined in oligonucleotide sequences) and ligated into NdeI/XhoI digested pET30a to generate an expression construct for a C-terminally His-tagged Vps4. The proteins were expressed in BL21 Rosetta cells and purified by chromatography over Ni-NTA resin (Qiagen). Rabbit antisera were generated against the recombinant proteins (Eurogentec). For immunolabeling on 50 nm sections, goat-anti-rabbit antibodies coupled to ultrasmall gold particles (Aurion, Wageningen, Netherlands) were used. Subsequently, samples were treated with a silver enhancement procedure (Danscher, 1981; Rachel et al., 2010).

## Results

### Overall cell anatomy

**Figure 1:**
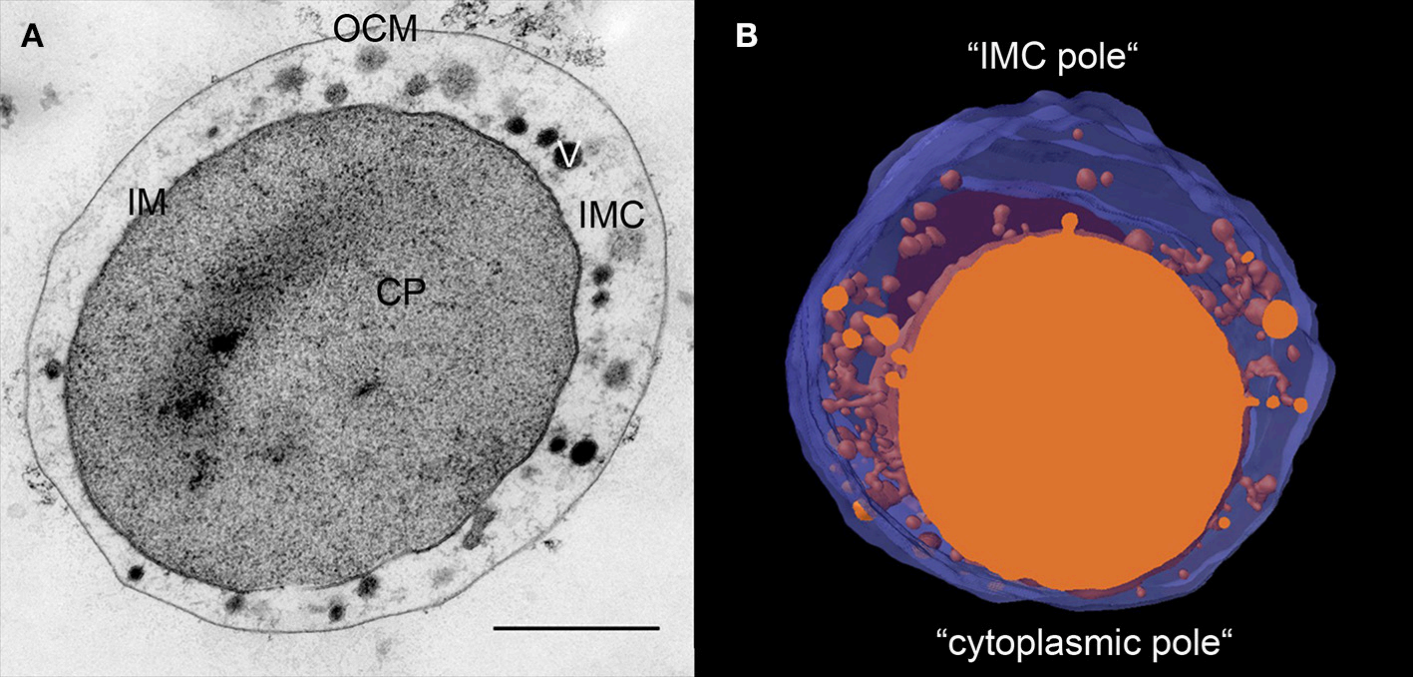
Ultrastructure of *Ignicoccus*. **(A)** 50 nm section of a high-pressure frozen, freeze-substituted *I. hospitalis* cell showing its characteristic constituents; CP, cytoplasm; IM, inner membrane; OCM, outer cellular membrane; IMC, inter-membrane compartment; V, vesicular structures; bar 0.5 μm; **(B)** 3D model of approximately half of an *I. hospitalis* cell based on 50 nm serial sections emphasizing the asymmetrical character of the cells; blue, OCM, orange cytoplasm and membrane surrounded structures in the IMC.

The ultrastructure of all *Ignicoccus* species as displayed in ultrathin sections is identical. In electron micrographs of 50 nm sections, the OCM and the IM are clearly visible as a double layer with two distinguishable electron dense layers for each membrane. These two electron dense layers as well as the layer in between are each 2–3 nm wide. Both membranes encase the IMC (Figure 1). From 3D models of whole *I. hospitalis* cells based on FIB/SEM and electron tomography, the volume of the IMC generally makes up ~40% of the whole cell volume. However, while most cells are 1–2 μm in diameter on average, we also observed cells of up to 5 μm for *I. hospitalis, I. islandicus*, and *I. pacificus* by light and/or electron microscopy. Cells of the strain MEX13A were even found to reach up to 20 μm. In these exceptionally larger cells, the volume of the IMC appears to be disproportionally larger and exceeds the volume of the cytoplasm.

Ultrathin sections and 3D models reveal that *Ignicoccus* cells develop poles in the cells (Figure 1). In every cell, there is a region where the IM comes to within 20–50 nm of the OCM. Notably, we observed that this is also the region where *Nanoarchaeum* cells are preferentially attached. The region on the opposite side shows a maximum distance between both membranes with a width of 200–500 nm, and sometimes even larger.

In contrast to the rather smooth OCM, the IM has a fluctuating, undulating structure and is different in shape from cell to cell. This is due to invaginations and protrusions of the cytoplasm. Although, 3D models based on 50 nm serial sectioning (Figure S1) suggest that free vesicles are present in the IMC, the resolution (100 nm in z, twice the section thickness) does not allow such an interpretation. Nevertheless, those models enabled preliminary 3D structures that were used as a foundation for higher resolution cellular reconstruction. To overcome the inherent limitations in resolution by 50 nm serial sectioning, we used FIB/SEM and electron tomography. Furthermore, we combined serial sectioning and electron tomography to cover large cell regions and also whole cells in detail.

### Endomembrane system

**Figure 2:**
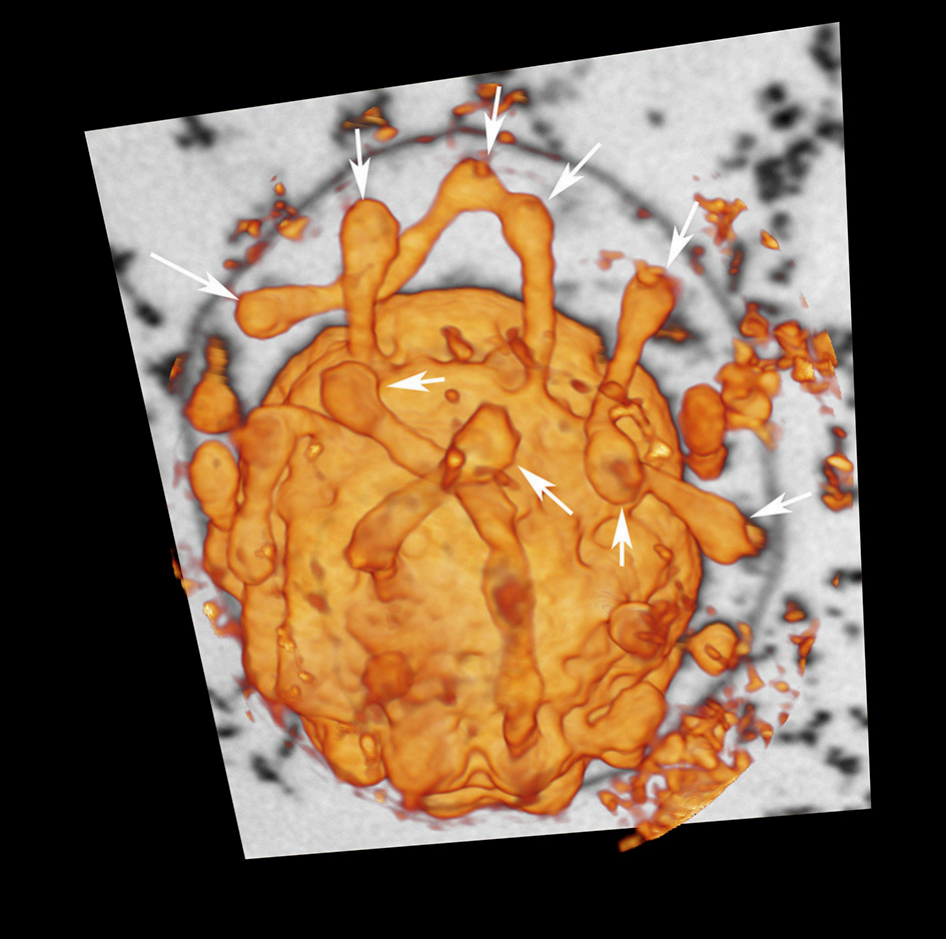
Cytoplasmic protrusions of *I. hospitalis*. “Voltex” (volume texture rendering) display of a *I. hospitalis* cell from the FIB/SEM data showing protrusions from the cytoplasm; arrows point to spherical swellings that might indicate constriction or fusion sites, respectively; additionally one slice of the image stack of the original data is shown, in which the OCM can be seen.

**Figure 3:**
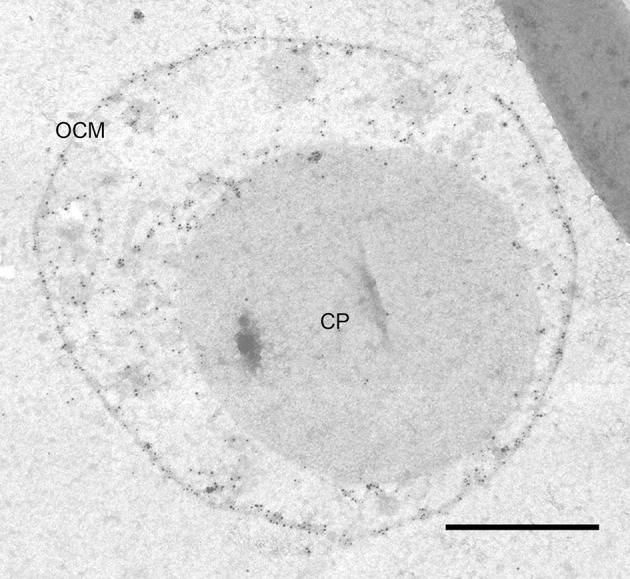
ATP synthase immunolabeling. Immunolabeling on 50 nm section of *I. hospitalis* against the A_1_ subcomplex of its ATP synthase; the labeling pattern shows localization in the OCM as well as in cytoplasmic protrusions; CP, cytoplasm; OCM, outer cellular membrane; bar 0.5 μm.

In contrast to previous descriptions of a vesicle transport system in *Ignicoccus* cells (Rachel et al., 2002), we observed very limited numbers of structures that could be interpreted as vesicles in the sense of individual membrane-surrounded compartments. Rather, most of the designated “vesicles” are contiguous with the cytoplasmic membrane and thus are cross-sections through protrusions of the cytoplasm (50–100 nm in diameter) or spherical swellings (150–180 nm in diameter) at their very ends (Figure 2, Videos S1–S4). Nevertheless, we found regions in the membranes of the bulk cytoplasm, the protrusions, and the swellings, which seem to be sites of constriction or fusion, respectively (Figure S2). For large cells of the strain MEX13A, we could show by phase contrast microscopy at 90°C that the inner membrane system indeed undergoes constant reorganization (Video S5). Electron micrographs also show that the protrusions or pseudovesicles are either textured like the cytoplasm or appear heavily stained and thus, presumably, are distinct in their composition. Moreover, in the course of earlier localization experiments of the archaeal A_1_A_*O*_ ATP synthase (Küper et al., 2010), we also obtained labeling patterns that show accumulation of the respective subcomplexes in cytoplasmic protrusions (Figure 3).

**Figure 4:**
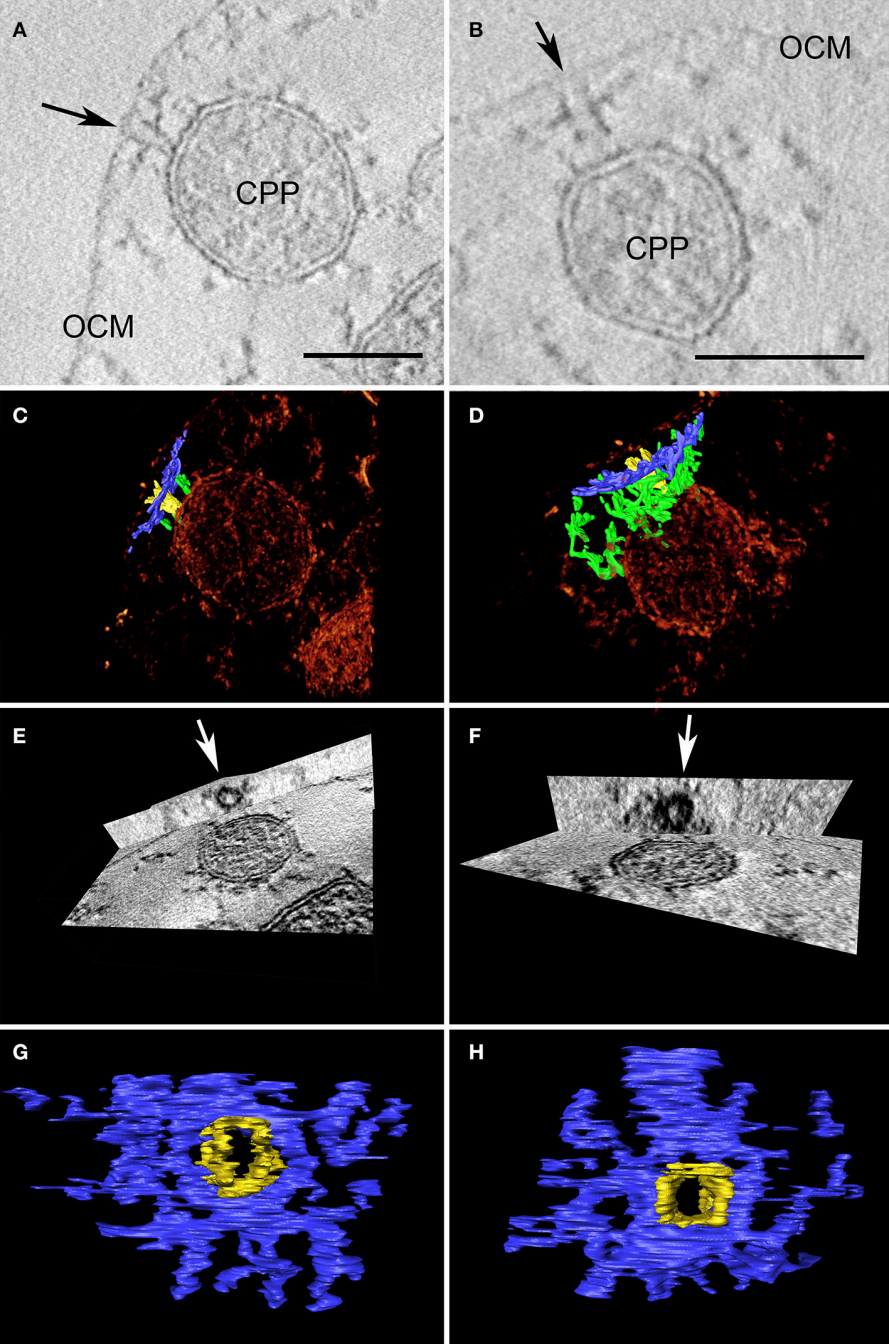
Two examples of cylindrical macromolecular complexes underneath the OCM. **(A,B)** Showing details of single slices of the respective tomograms, arrow points to the ending of the cylindrical complex at the OCM; OCM, outer cellular membrane; CPP, cytoplasmic protrusion; bar 50 nm each; **(C,D)** combined view of the respective sub-volumes, showing segmented macromolecular complexes (blue and yellow) in a volume texture rendering display, additional filamentous structures are labeled in green; **(E,F)** show cross-sections of the subvolumes with “ObliqueSlice” tool; orthogonal to the xy plane an oblique plane was chosen that cuts through the structure “parallel” to the OCM; arrow points to the inner “ring” of the structure; **(G,H)** top view of segmented macromolecular complexes; note that the yellow and blue parts serve as a color coding for what is coming out of/going through the plane.

We emphasize that we have never detected similar constriction or fusion sites between cytoplasmic protrusions and the OCM in the course of structural studies on *Ignicoccus* isolates over the past 10 years. However, we detected structures that can be regarded as complexes mediating putative interactions between these two membrane systems. Approximately 20 nm underneath the OCM (but still in contact with it), dark contrasted elongated structures with a length of about 50–100 nm were observed in ultrathin sections (Figure S3). Such structures can be seen in regions where cytoplasmic protrusions are close to the OCM, but they were also found between the “core”-cytoplasm and the OCM. A perpendicular structure to the electron-dense structure at the OCM can be observed on top of the inner membrane component, thus connecting the two membranes indirectly. These structures were analyzed in detail in tomograms of tilt series: once more, the 3D approach showed its full potential as the dark lines underneath the OCM were found to be cylindrical structures with an inner diameter of ~20 nm and an outer diameter of ~100 nm (Figure 4).

### Filamentous matrix in the IMC

**Figure 5:**
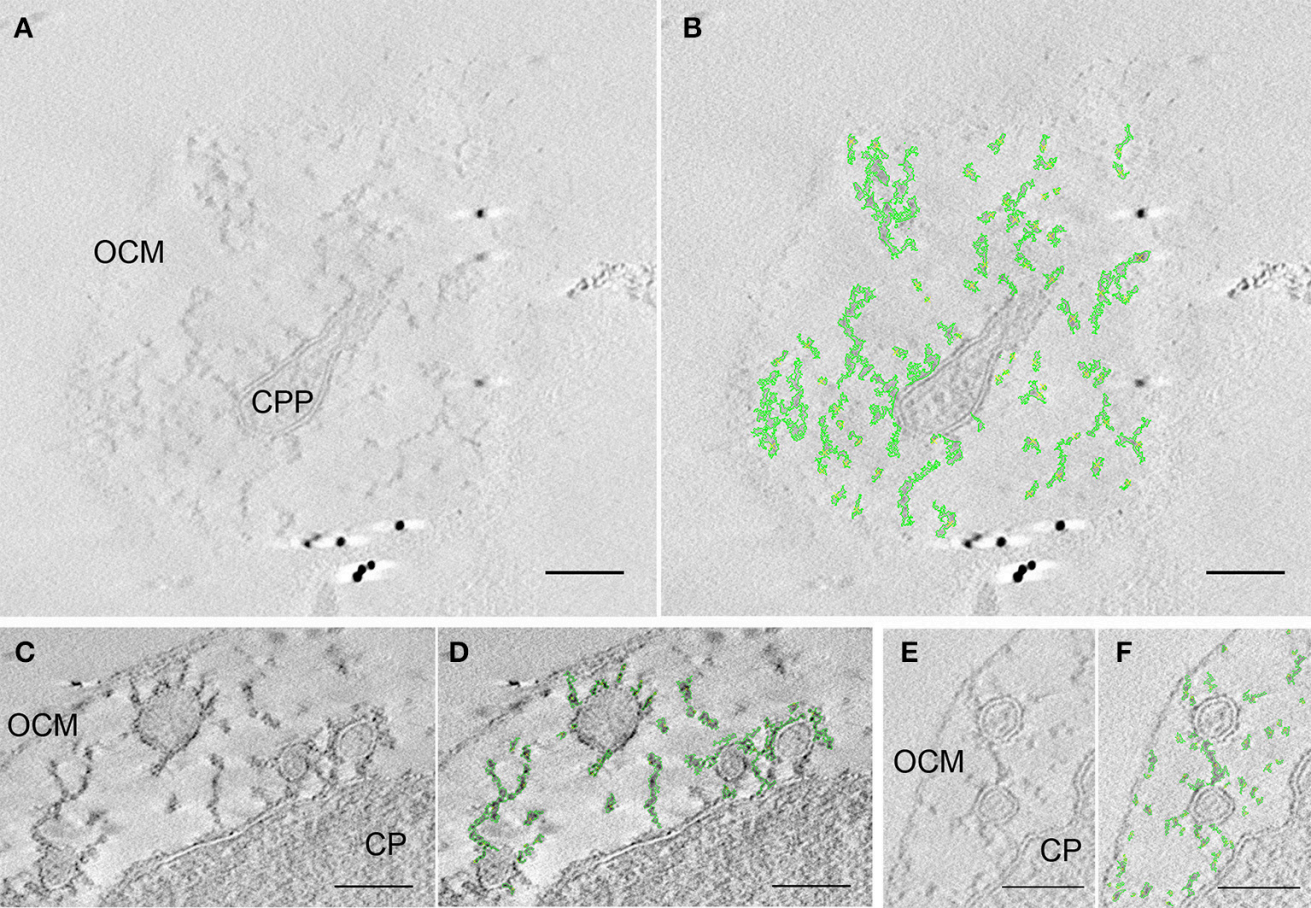
Matrix of filaments and/or thethers in the IMC. Three pairs of sections of tomograms each showing the original data **(A,C,E)** and the respective structures highlighted in green **(B,D,F)**; **(A,B)** is taken from an edge region of a tomogram that shows a cytoplasmic protrusion and its connection via the branched matrix to the OCM; **(C,D)** as well as **(E,F)** show examples of interconnected protrusions and connection between the cytoplasm and protrusions, a connection between the protrusions and the outer cellular membrane can also be seen; OCM, outer cellular membrane; CP, cytoplasm; CPP, cytoplasmic protrusion; bars 100 nm, each.

In the IMC, filamentous structures of 3–6 nm in diameter were observed. These hitherto undocumented structures are visible in almost all electron micrographs of Epon-embedded *Ignicoccus* cells. Figure 5 shows examples of how these structures look in tomograms. The filaments are not straight, but appear more like springs or chains, and build up a branched network that spans through the whole IMC. In 3D reconstructions, this interconnecting function becomes even more evident and the structures appear to be the main constituent visible inside the IMC, linking all membrane-surrounded components with themselves and the OCM (Videos S3, S4, S6). Considering the dynamics of the endomembrane system (Video S5) and the fact that the ATP synthase is located in the OCM, which presumably makes a considerable amount of ATP available in the IMC, the filamentous structures might act as tethers and/or as a cytoskeleton.

**Figure 6:**
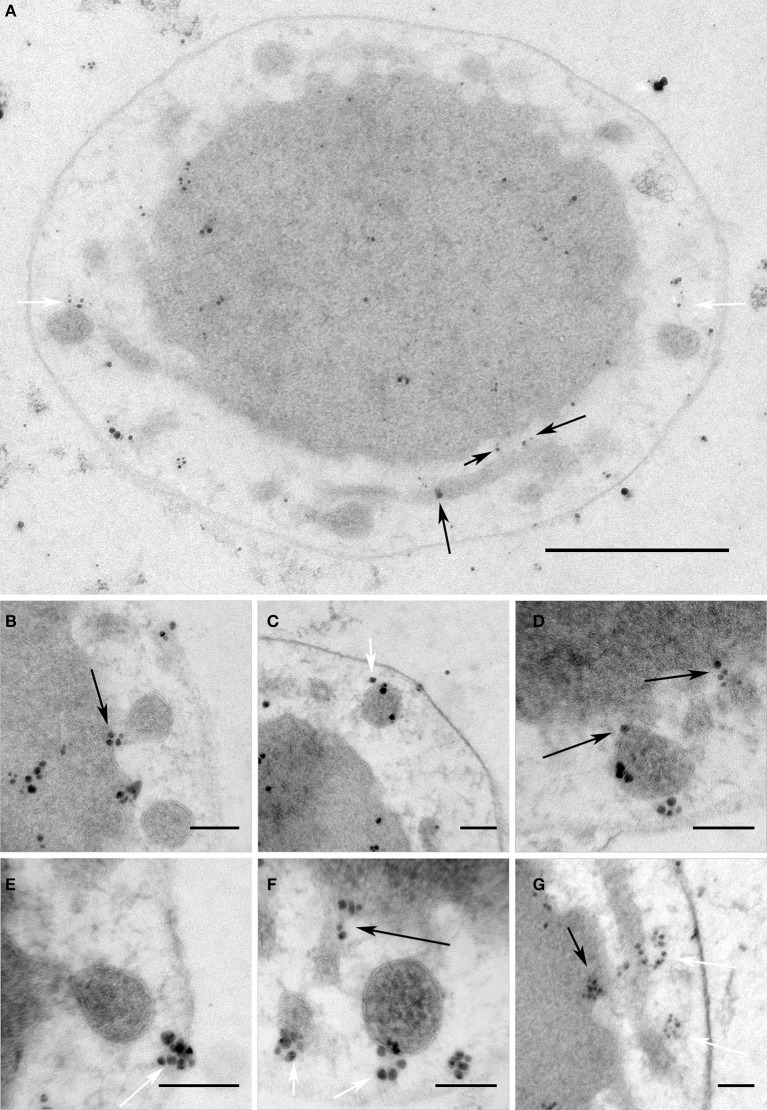
V4R protein Igni_1332 immunolabeling. Immunolabeling on 50 nm sections against Igni_1332 showing **(A)** a whole *I. hospitalis* cell and **(B–G)** additional examples from different *I. hospitalis* cells; black arrows point to examples for localizations in the IM and the membrane of protrusions at putative fusing sites; white arrows point to labeling associated with the matrix of filaments and/or tethers in the IMC; bar 0.5 μm **(A)**; 100 nm **(B–G)**

Interestingly, *I. hospitalis* possesses seven proteins with a 4-vinyl reductase (V4R) domain. This V4R domain is homologous to Bet3, a component of the TRAPP I vesicle tethering complex in Eukaryotes (Podar et al., 2008b). In Eukaryotes, these complexes “catch” vesicles and bring them close to the target membrane. Subsequently, SNARE proteins of both membranes can interact and initiate the membrane fusion process (for details see Cai et al., 2007). By immunolabeling, we could detect that one of the V4R proteins in *I. hospitalis* (Igni_1332) is located in the IM (including the membrane of protrusions) and associated with the matrix in the IMC. Moreover, labeling at putative fusion sites (regions where membranes approach one another) could be frequently observed (Figure 6).

### Vacuole-like compartment

Very rarely, we observed additional, vacuole-like compartments. These compartments are 300–400 nm in diameter and are surrounded by a pentalamellar membrane (three electron-dense and two electron-lucent layers). Except for a few filaments, no other structures were observed inside. Videos S3, S4, and Figure S4 show the only documented examples, so far.

### Phosphate storage

**Figure 7:**
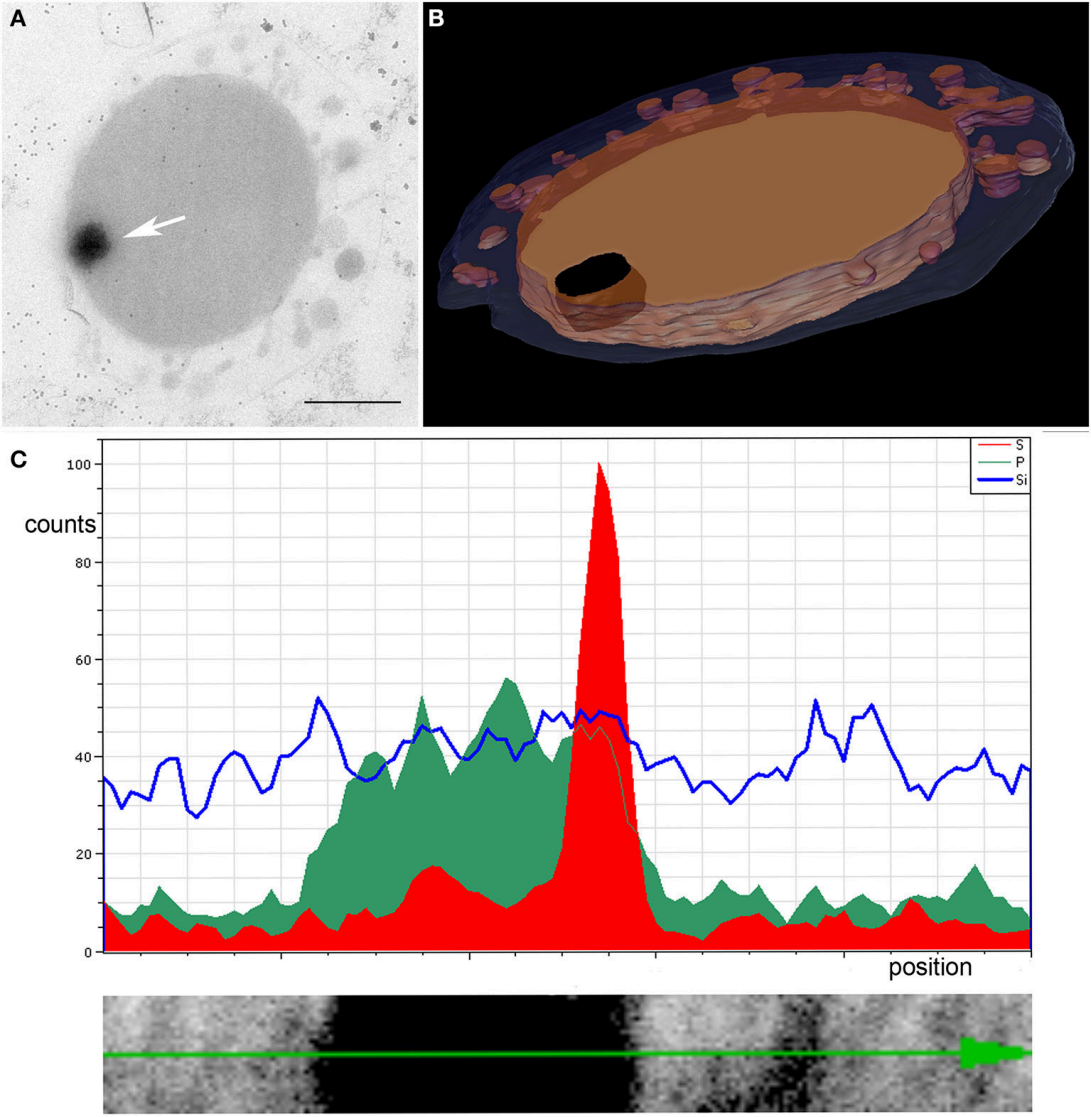
EDX analysis of dark contrasted inclusion in the cytoplasm. **(A)** Arrow points to the structure in a slice of a tomogram, bar 0.5 μm; **(B)** shows the 3D model of the 200 nm section; blue, OCM; orange, cytoplasm and cytoplasmic protrusions; black, inclusion in the cytoplasm; **(C)** linescan across the structure showing accumulation of phosphorus (green) inside the structure and a sulfur peak (red) at its edge

Frequently, one or more dark contrasted inclusions of 80–250 nm in diameter were observed in the cytoplasm of *Ignicoccus* cells, in close proximity to the IM. Energy dispersive X-ray analysis (EDX) revealed a high content of phosphorus inside these inclusions (Figure 7). Since uranium was also found in high amounts (which was used in the form of uranyl acetate as a contrasting agent and has a high affinity to phosphate compounds), these inclusions can be considered as phosphate storage, which is quite common among prokaryotic and eukaryotic cells. Interestingly, we observed a sulfur peak at the edge of this structure. A mapping analysis confirmed this and also showed iron in this region, thus suggesting the presence of iron-sulfur-cluster proteins in this edge region of the structure (Figure S5).

### Contact site and protein transfer to *Nanoarchaeum equitans*

**Figure 8:**
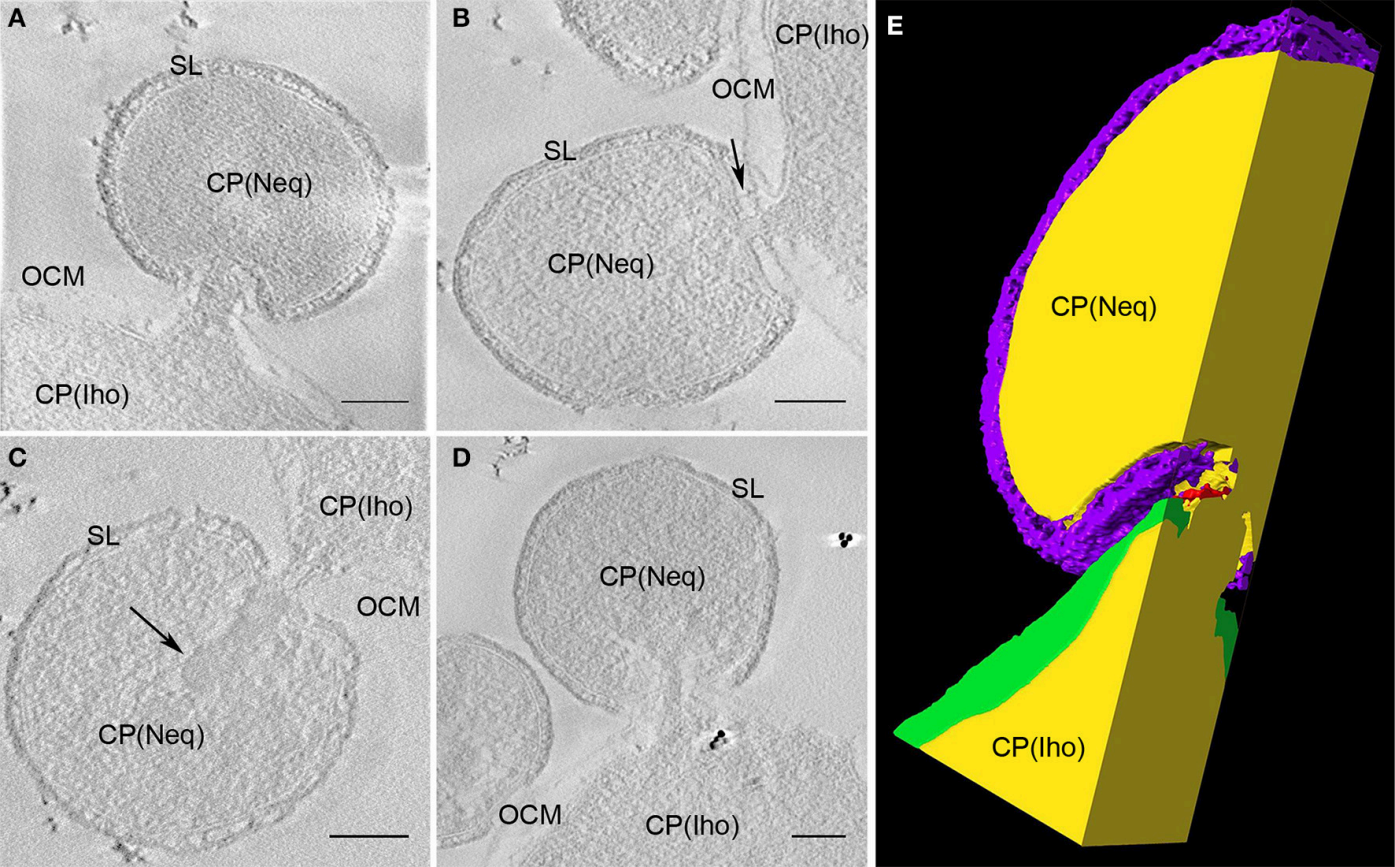
Contact site of *N. equitans*. **(A–D)** Show slices of tomograms in which the direct connection between the cytoplasms of both organisms can be followed; in each example the cytoplasm of *I. hospitalis* protrudes toward *N. equitans*, which appears to pierce both *I. hospitalis* membranes via a stalk like structure, the S-Layer appears to be disintegrated in the contact region; in **(B)** also *N. equitans* tethers can be seen (arrow), that were already described before; in **(C)** the arrow points to an electron dense structure of unknown composition and function in the cytoplasm of *N. equitans* at the basis of the stalk OCM, outer cellular membrane; SL, S-Layer; CP, cytoplasm; Iho, *I. hospitalis*; Neq, *N. equitans*; bar 100 nm, each; **(E)** cross section of the 3D model of the sub-volume shown in **(A)**; purple, S-Layer of *N. equitans*; green, OCM of *I. hospitalis*; yellow, cytoplasms of both organisms; red, presumeably either filaments from *I. hospitalis* or parts of the disintegrated S-Layer of *N. equitans*.

Next, we aimed to determine the nature of the interaction between *I. hosptalis* and *N. equitans*. Strikingly, our data reveal that the cytoplasms of both organisms get into direct contact. As can be seen in Figure 8 and Video S7, a part of the cytoplasm of *I. hospitalis* protrudes toward a cell of *N. equitans*. This protrusion is most prominent in Figure 8A and is enclosed by *N. equitans* at a site where the S-Layer is not visible, suggesting that the S-layer is disintegrated in the contact region. In all of the examples shown, *N. equitans* exhibits a small stalk of ~30 nm in diameter (~20 nm, if measured without the membranes) that appears to pierce the OCM and the IM of *I. hospitalis*, thus leading to a direct connection of cytoplasms of both organisms. In Figure 8C, electron dense material of unknown composition and function can be seen at the origin of the stalk. In addition, in Figure 8A filamentous structures on top of the endomembrane system of *I. hospitalis* seem to be involved in building up this contact site, as well as tethers of *N. equitans* that can be seen in Figure 8B; such tethers were already described in earlier studies (Junglas et al., 2008). The example in Figure 8A was also modeled; Figure 8E is a cross section through the model showing a plane in which the cytoplasmic bridge can be seen. The model was also visualized in a video (Video S7). Further details on the contact site could be revealed by cryo electron microscopy (ongoing studies).

**Figure 9:**
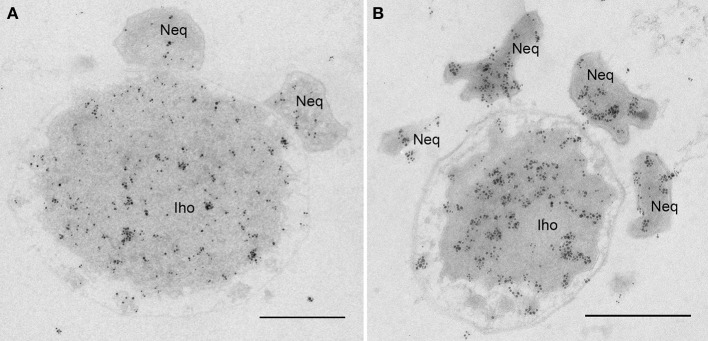
Protein transfer from *I. hospitalis* to *N. equitans*. Micrographs show immunolabelings on 50 nm sections of *I. hospitalis* proteins **(A)** Vps4 (Igni_0994) and **(B)** a fatty acid coA ligase (Igni_0475); in both cases proteins are found to be distributed in the cytoplasm of both organisms, although no homologs are annotated for *N. equitans*; Iho, *I. hospitalis*; Neq, *N. equitans*; bars, 0.5 μm each.

Based on the observed cytoplasmic connection, we aimed to determine whether protein transport from *I. hospitalis* to *N. equitans* may occur. Indeed, by immunolabeling using specific antibodies, we were able to detect *I. hospitalis* proteins (Igni_0994—Vps4; Igni_0475—fatty acid coA ligase) in the cytoplasms of both organisms (*I. hospitalis* and *N. equitans*) when *N. equitans* is attached (Figure 9). Importantly, no homologs of these *I. hospitalis* proteins are encoded by the genome of *N. equitans*.

## Discussion

### A complex and dynamic endomembrane system in *I. hospitalis* with putative secretory function

Even among Archaea with two membranes (see Klingl, 2014), the ultrastructure of *I. hospitalis* is unique. *Ignicoccus* cells are asymmetrical and possess a complex endomembrane system. Moreover, we could visualize an interconnecting matrix of filaments and/or tethers in the voluminous IMC. We suggest that this matrix could be involved in dynamic processes, i.e., movement and constriction and/or fusion processes of cytoplasmic protrusions with each other and with the bulk cytoplasm. Since the ATP synthase is located in the OCM (Küper et al., 2010), considerable amounts of ATP are presumably available in the IMC to deliver the necessary energy for such dynamic processes. Based on our immunolabeling studies that showed a localization of the A_1_ subcomplex in cytoplasmic protrusions, it seems likely that proteins are transported to the OCM via cytoplasmic protrusions. In addition, import of ATP from the OCM might be taken into consideration. ATP is generated in the IMC but needs to be transported to the cytoplasm to provide the necessary cellular energy. In this context, it is also noteworthy that we did not only find cytoplasmic protrusions that are textured like the cytoplasm, but also highly contrasted ones. As the contrast agent uranyl acetate has a high affinity for carboxy- and phosphate groups, the dark protrusions might depict a “loaded state” with proteins and/or ATP. For such a transport process, the cylindrical complexes in the OCM could act as “docking sites” for cytoplasmic protrusions. Presumably, the cylindrical complexes are the same structures previously observed in freeze-etching samples of *I. hospitalis* cells, and named “24 nm pores” (Rachel et al., 2002). A recent study suggests that these complexes are involved in secretion and thus also in assembly of *I. hospitalis* fiber proteins. Notably, a cylindrical macromolecular complex can be seen on the site where the fiber of *I. hospitalis* originates (see Figure 3D in Meyer et al., 2014).

### A direct cytoplasmic connection between *N. equitans* and *I. hospitalis*

Regarding *N. equitans*, we almost exclusively observed contact sites in regions where either the bulk cytoplasm, or cytoplasmic protrusions, were near the OCM in *I. hospitalis*. This gave a first hint on the involvement of the endomembrane system of *I. hopitalis* in establishing the contact between the two organisms. It has been proposed that tethers of *N. equitans* and the *N. equitans* S-Layer play a role in the initial contact between the cells (Junglas et al., 2008; Burghardt et al., 2009). However, when establishing the direct cytoplasmic connection, the *N. equitans* S-Layer is disintegrated at the actual contact region. *N. equitans* appears to pierce the OCM utilizing a stalk-like structure of 20–30 nm in diameter, and thus tap the endomembrane system of *I. hospitalis*. This disrupting effect on the *I. hospitalis* cell is also supported by comparative proteomic analysis correlating a higher expression of mechanosensitive channels (Igni_0056 and Igni_0235) in *I. hospitalis* with an increasing number of attached *N. equitans* cells (Giannone et al., 2015). A direct cytoplasmic connection between the two organisms was previously proposed based on live/dead cell staining, in which attached *N. equitans* cells exhibited the same color as their host cell (Jahn et al., 2008). In the same study, it was concluded that amino acids are transported from *I. hospitalis* to *N. equitans*. This is now supported by a recent metabolomic study suggesting that a pool of metabolites, including ATP, might be taken up by *N. equitans* (Hamerly et al., 2015). In addition, proteomic analysis of purified *N. equitans* cells revealed several *I. hospitalis* proteins (Giannone et al., 2011). In the context of a direct cytoplasmic connection, it seems likely that at least some of these *I. hospitalis* proteins were taken up by *N. equitans*. Indeed, by immunolabeling on ultrathin sections, we could detect two *I. hospitalis* proteins in *N. equitans* for which no respective homologs are annotated in the *N. equitans* genome. We note that the eukaryotic ortholog of one of these proteins, Vps4 (Igni_0994), plays a pivotal role in the generation of intralumenal vesicles in the ESCRT pathway of eukaryotic endosomal sorting. Archaeal ESCRT proteins have been implicated in cell division and vesicle generation (Lindås et al., 2008; Samson et al., 2008; Ellen et al., 2009). While it is likely that the ESCRT machinery is involved in cell division in *Ignicoccus, N. equitans* may employ its FtsZ homolog for cytokinesis (Makarova et al., 2010). Thus, we speculate that the Vps4 we observed within *N. equitans* may be associated with transport processes. The other *I. hospitalis* protein that we found in *N. equitans* (Igni_0475, a fatty acid coA ligase) might complement the limited biosynthesis capabilities of *N. equitans*. However, it is also possible that both proteins just undergo metabolic degradation. In any case, acquiring energy (in the form of ATP), additional metabolites, and proteins from its host enables *N. equitans* to proliferate. Moreover, in contrast to previous suggestions (Huber et al., 2012), a sophisticated transport system spanning two or three membranes does not appear necessary for this. A cytoplasmic connection between *I. hospitalis* and *N. equitans* would also facilitate lateral gene transfer, which was suggested to have occurred in both directions (Podar et al., 2008a). Presumably, *I. hospitalis* cells ultimately collapse and die. However, this suggestion is based on laboratory observations that could allow other interpretations as well. In co-cultures at stationary phase, many unbound *N. equitans* cells, but hardly any unoccupied *I. hospitalis* cells can be detected. This may indicate that some proliferating *N. equitans* cells detach and are “searching” for a new host cell. If no host cells are available, those detached *N. equitans* cells eventually die. However, re-attaching of free *N. equitans* to *I. hospitalis* cells has not yet been demonstrated. Also, some electron micrographs can be interpreted as showing remnants of dying/lysed *I. hospitalis* cells surrounded by several intact *N. equitans* cells (Figure S6). Thus, this would characterize *N. equitans* as a parasitic or parasitoid organism (Eggleton and Gaston, 1990), supported by the fact that no benefit for *I. hospitalis* in this relationship has been shown, so far.

### An archaeal origin of the eukaryotic endomembrane system?

Currently, it is widely accepted that eukaryotic cells emerged from an archaeal host and a bacterial endosymbiont. However, the origin of the eukaryotic endomembrane system remains an enigma. In our study we could show that a complex endomembrane system does exist within Archaea—i.e., at least in *I. hospitalis*—supporting hypotheses that suggest an archaeal origin of the eukaryotic endomembrane system. This is in agreement with phylogenetic analyses that place *Ignicoccus* as a member of the TACK superphylym (Guy and Ettema, 2011), hypothesized to be a sister group of the recently described Asgard archaea and Eukaryotes (Lake et al., 1984; Cox et al., 2008; Foster et al., 2009; Kelly et al., 2011; Williams et al., 2012, 2013; Wolf et al., 2012; Yutin et al., 2012; Lasek-Nesselquist and Gogarten, 2013; Martijn and Ettema, 2013; Koonin and Yutin, 2014; Williams and Embley, 2014; Petitjean et al., 2015; Zaremba-Niedzwiedzka et al., 2017). In TACK archaea, several “eukaryotic signature proteins” (ESPs) are present including homologs to proteins that are involved in membrane remodeling processes in Eukaryotes (Guy and Ettema, 2011). For Asgard archaea, this set of ESPs has been shown to be expanded (Zaremba-Niedzwiedzka et al., 2017). It is possible, therefore that the last common ancestor of archaea and Eukaryotes might already have had the ability to bend membranes and form internal vesicles (Zaremba-Niedzwiedzka et al., 2017).

It should be noted that bacterial complex endomembrane systems are also known to exist. They have been described for some genetically manipulated organisms (e.g., Weiner et al., 1984; Maklashina et al., 1998; Lefman et al., 2004) and members of the PVC phylum. Most notably, the planctomycete, *Gemmata obscuriglobus*, reveals some striking similarities to *Ignicoccus*: an extended periplasm between the inner and outer membrane, interconnected vesicle-like structures that build a continuum with the cytoplasm, the presence of highly contrasted vesicles, and contact sites to the outer membrane. (Santarella-Mellwig et al., 2013; Acehan et al., 2014). As the Planctomycete endomembrane system might represent an analogous structure that arose independently, it is not necessarily in conflict with an archaeal origin of the eukaryotic endomembrane system. But, a putative archaeal origin of the eukaryotic endomembrane system is in contrast with a more recent hypothesis suggesting that the eukaryotic endomembrane system originates from outer membrane vesicles released from the α-proteobacterial symbiont (mitochondrial ancestor) in an archaeal host (Gould et al., 2016). However, this does not contradict the idea that fusion events might have happened between such putative (bacterial) outer membrane vesicles and an already complex (archaeal) endomembrane system.

## Conclusions

In our study, we reveal that *I. hospitalis* possesses a complex endomembrane system with extensive cytoplasmic protrusions. These protrusions were found to interact with themselves and the IM in a highly dynamic manner for which a filamentous network of yet unknown composition in the IMC might play a role. We also found interaction of the cytoplasm and protrusions with the OCM via cylindrical macromolecular complexes, suggesting a secretory function for this endomembrane system.

Observations on the contact site to *N. equitans* revealed that no sophisticated transport system spanning two or three membranes for proteins/metabolites/DNA is necessary. The S-layer is disintegrated at the contact region and the OCM of *I. hospitalis* is disrupted. Thus, the cytoplasms of both organisms can connect. In consideration of recent ultrastructural, proteomic, transcriptomic, metabolomic, and cultivation studies, our data support the view, that *N. equitans* represents a parasitic or parasitoid organism.

This study, in addition to the recent findings of ESPs related to eukaryotic trafficking machineries in TACK and Asgard archaea, supports an archaeal origin of the eukaryotic endomembrane system.

## Author contributions

TH, JF, SB, HH, RW, MP, and RR planned the experiments. TH, JF, and CP cultivated the organisms, prepared genomic DNA, prepared any samples for electron microscopy and performed the immunolabeling experiments. RS, SB, LW, and MP performed cloning, protein purification, and generation of antibodies. HH and RW acquired thermo microscopy movies. TH, SG, and RR acquired TEM tilt series. GW acquired the FIB/SEM data. TH, CP, and JZ performed the EDX analysis. TH, JF, VH, and BS reconstructed and visualized the 3D data. TH wrote the manuscript with input from JF, SB, HH, MP, and RR.

### Conflict of interest statement

The authors declare that the research was conducted in the absence of any commercial or financial relationships that could be construed as a potential conflict of interest.

## Abbreviations

ESP: eukaryotic signature protein
FIB/SEM: focused ion beam scanning electron microscopy
IM: inner membrane
IMC: inter-membrane compartment
OCM: outer cellular membrane
V4R: 4-vinyl reductase.
