# Supplementary material for: A Complex Endomembrane System in the Archaeon Ignicoccus hospitalis Tapped by Nanoarchaeum equitans
Source: Front Microbiol. 2017 Jun 13;8:1072. doi: 10.3389/fmicb.2017.01072 (PMC5468417; doi:10.3389/fmicb.2017.01072)
Supplement: Supplementary file 8 [file DataSheet1.DOCX]

Supplementary Material

A Complex Endomembrane System in the Archaeon *Ignicoccus* *hospitalis* Tapped by *Nanoarchaeum equitans*

T Heimerl^*^, J Flechsler, C Pickl, V Heinz, B Salecker, J Zweck, G Wanner, S Geimer, RY Samson, SD Bell, H Huber, R Wirth, L Wurch, M Podar, R Rachel

*** Correspondence:** Thomas Heimerl: thomas.heimerl@synmikro.uni-marburg.de

# Supplementary Figure Captions

**Fig S1. 3D models of *I. hospitalis* based on 50 nm serial sections.**

36 3D models of *I. hospitalis* based on 4-28 sections; interpolation was done to smoothen the surfaces**;** blue OCM, orange cytoplasm and membrane surrounded structures; note that the shape of the inner membrane system looks different in each cell and that a polarization of the cells is apparent

**Fig S2. Putative constriction/fusion sites of inner membrane system components.**

20 examples from ultrathin sections of *I. hospitalis* of putative constriction/fusion sites; bars, 100 nm

**Fig S3. "Docking sites" of protrusions at the OCM as seen in 50 nm sections.**

note the "dark lines" underneath the OCM and a perpendicular structure originating from the inner membrane system towards these "dark lines"

**Fig S4. Vacuole like structures in *I. hospitalis*.**

(A) ultrathin section, (B) slice of a tomogram of *I. hospitals* with detail showing the pentalamellar character of the structure; the 3D model of the cell in (B) and the tomogram are presented in Video S3 and S4, respectively; bars 0.5 µm

**Fig S5. EDX mapping analysis.**

(A) shows a dark (electron-dense) structure in the cytoplasm of an *I. hospitalis* cell in a bright field image, and the distribution of the elements (B) phosphor, (C) iron, (D) sulfur in this structure; bar: 70 nm

**Fig S6. *N. equitans* is likely to be a parasitoid organism**.

6 consecutive 50 nm sections showing several *N. equitans* cells surrounding putative remnants of an *I. hospitalis* cell; bars, 0.5 µm

# Supplementary Video Captions

**Video S1. Original data stack and 3D model of *I.hospitalis* cells based on FIB/SEM.**

Covered volume 30 µm^3^ with a voxel size of 5 x 5 x 5 nm; OCM of the cells is shown in a transparent view, the cytoplasm and its protrusion are opaque

**Video S2. 3D model of *I.hospitalis* cells based on FIB/SEM.**

Covered volume 386 µm^3^ with a voxel size of 5 x 5 x 10 nm, OCM of the cells is shown in a transparent view, the cytoplasm and its protrusion are opaque

**Video S3. 3D model of whole *I. hospitalis* cell.**

The video shows a 3D model of *I. hospitalis* based on 9 consecutive tomograms of 200 nm sections (1278 single pictures); orange, inner membrane system comprising the cytoplasm and its protrusions; red, a vacuole-like structure in the IMC; green, filamentous structures in the IMC; blue, OCM; the respective tomogram is shown in Video S4

**Video S4. Joined tomogram of a whole *I. hospitalis* cell.**

Stack of 9 consecutive tomograms of 200 nm sections, 1278 single pictures on which Video S3 is based on

**Video S5. Dynamics in strain MEX13A.**

Phase contrast microscopy at physiological temperature of 90°C shows a constant reorganization of the inner membrane system in the cell in real time

**Video S6. Detail of filamentous interconnecting structures in the IMC of *I. hospitalis*.**

The video shows a tomogram of a 200 nm section of *I. hospitalis*, a subvolume was modeled in 3D; blue, OCM; orange, cytoplasm and protrusions; green, interconnecting filamentous structures in the IMC

**Video S7. Detail of the contact site of *N.equitans* to *I. hospitalis*.**

Tomogram and 3D model of a subvolume of a 200 nm section are shown (see also Fig 7); yellow, cytoplasms of both organisms; green, IMC; purple, S-Layer of N.equitans; red, presumeably either filaments from *I. hospitalis* or parts of the disintegrated S-Layer of *N. equitans*
