# Supplementary figures and images for: A Complex Endomembrane System in the Archaeon Ignicoccus hospitalis Tapped by Nanoarchaeum equitans
Source: Front Microbiol. 2017 Jun 13;8:1072. doi: 10.3389/fmicb.2017.01072 (PMC5468417; doi:10.3389/fmicb.2017.01072)

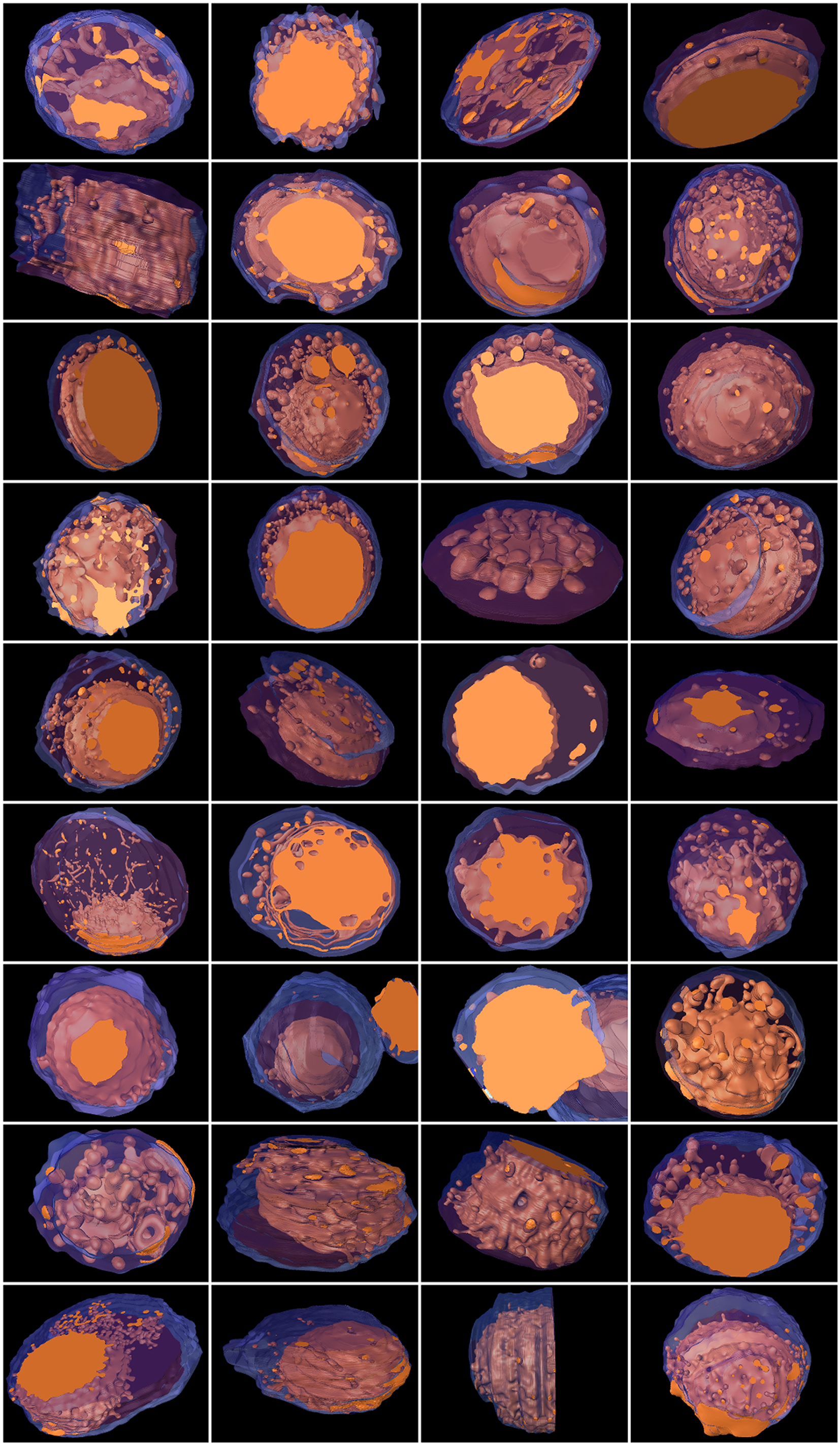

Supplement: Supplementary file 9 [file Image1.TIF]

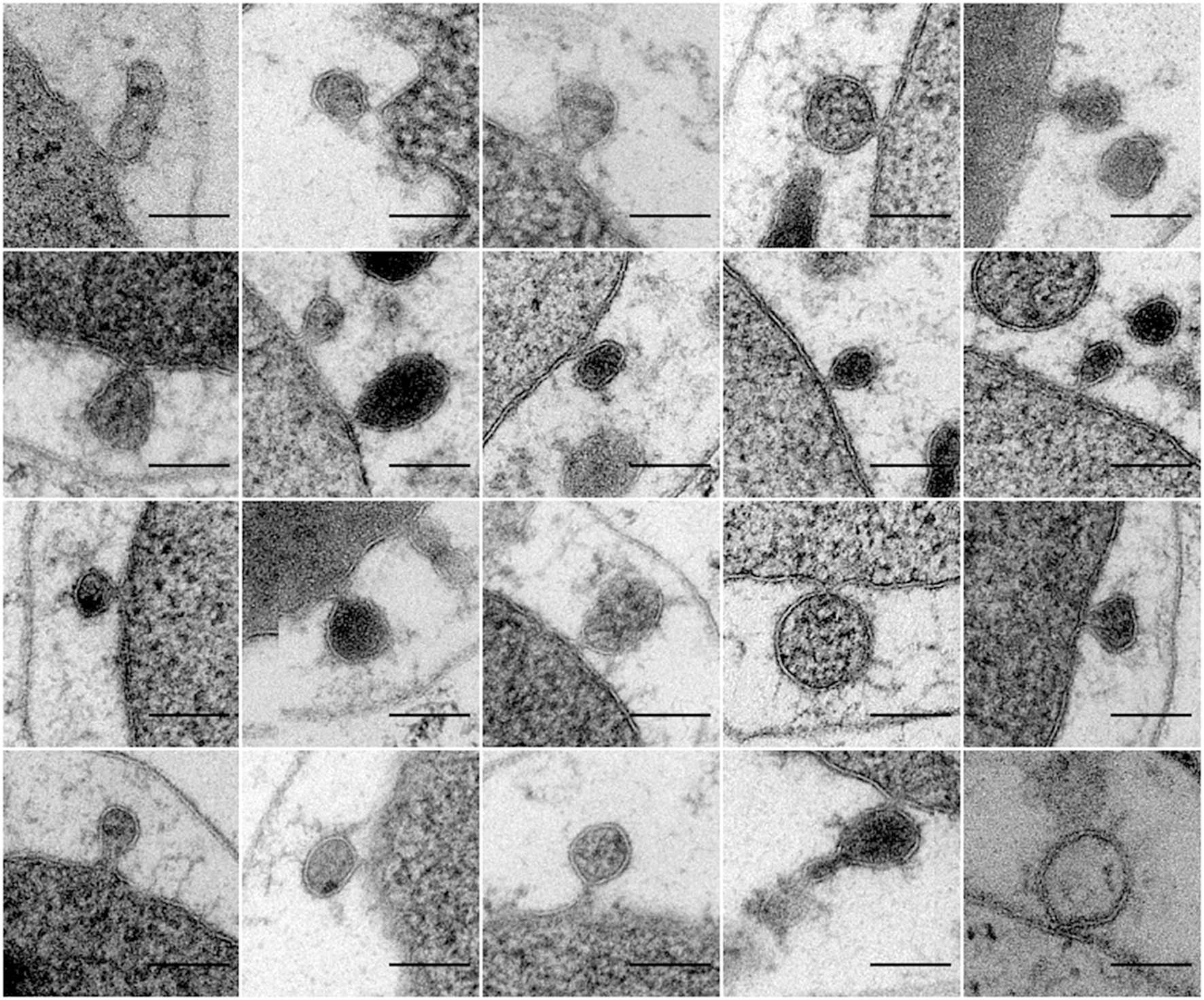

Supplement: Supplementary file 10 [file Image2.TIF]

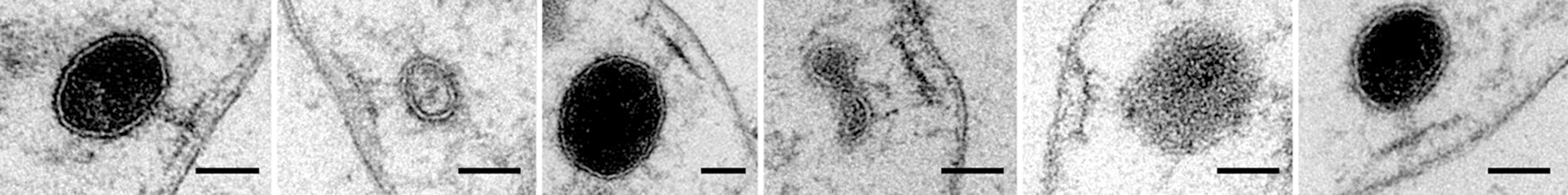

Supplement: Supplementary file 11 [file Image3.TIF]

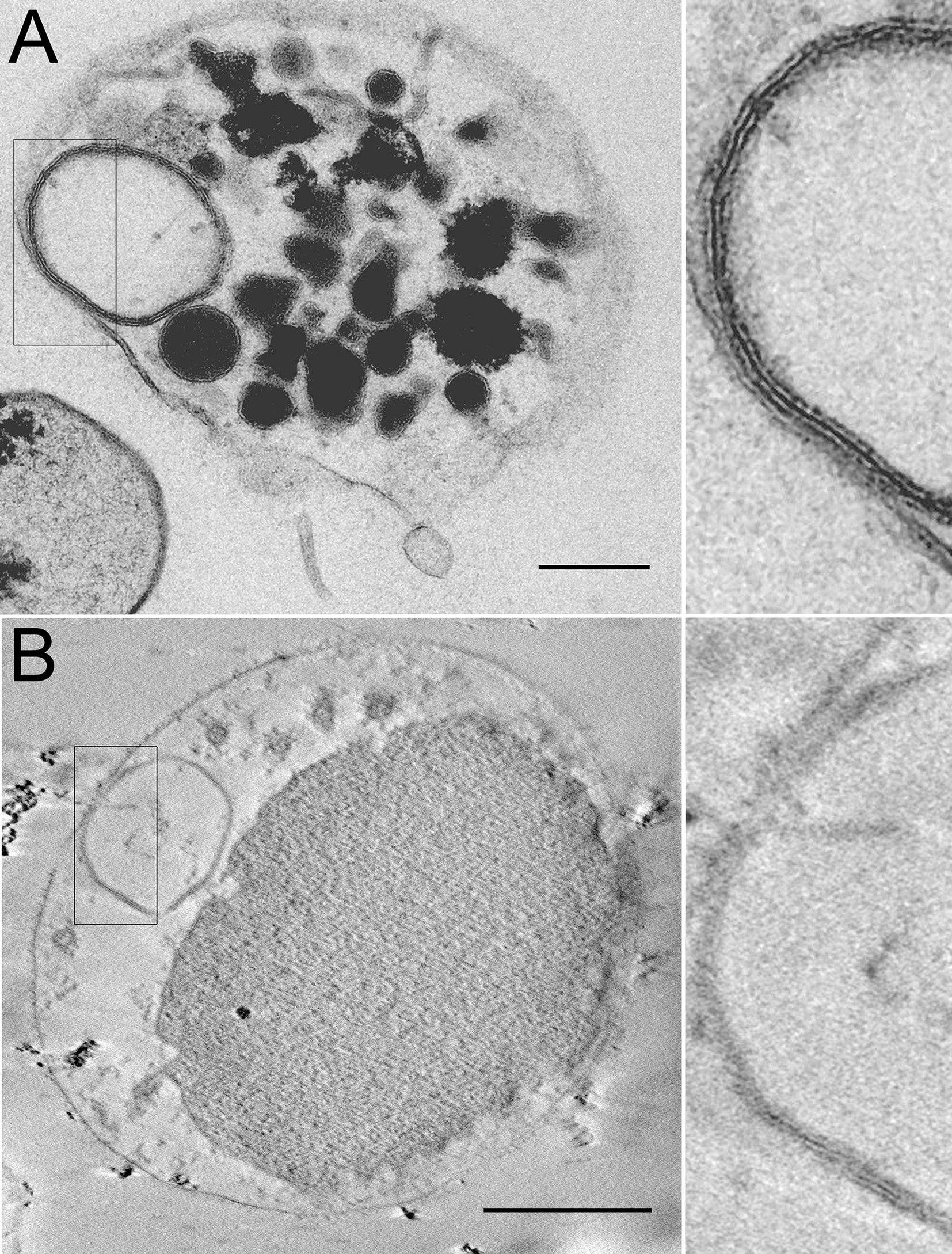

Supplement: Supplementary file 12 [file Image4.TIF]

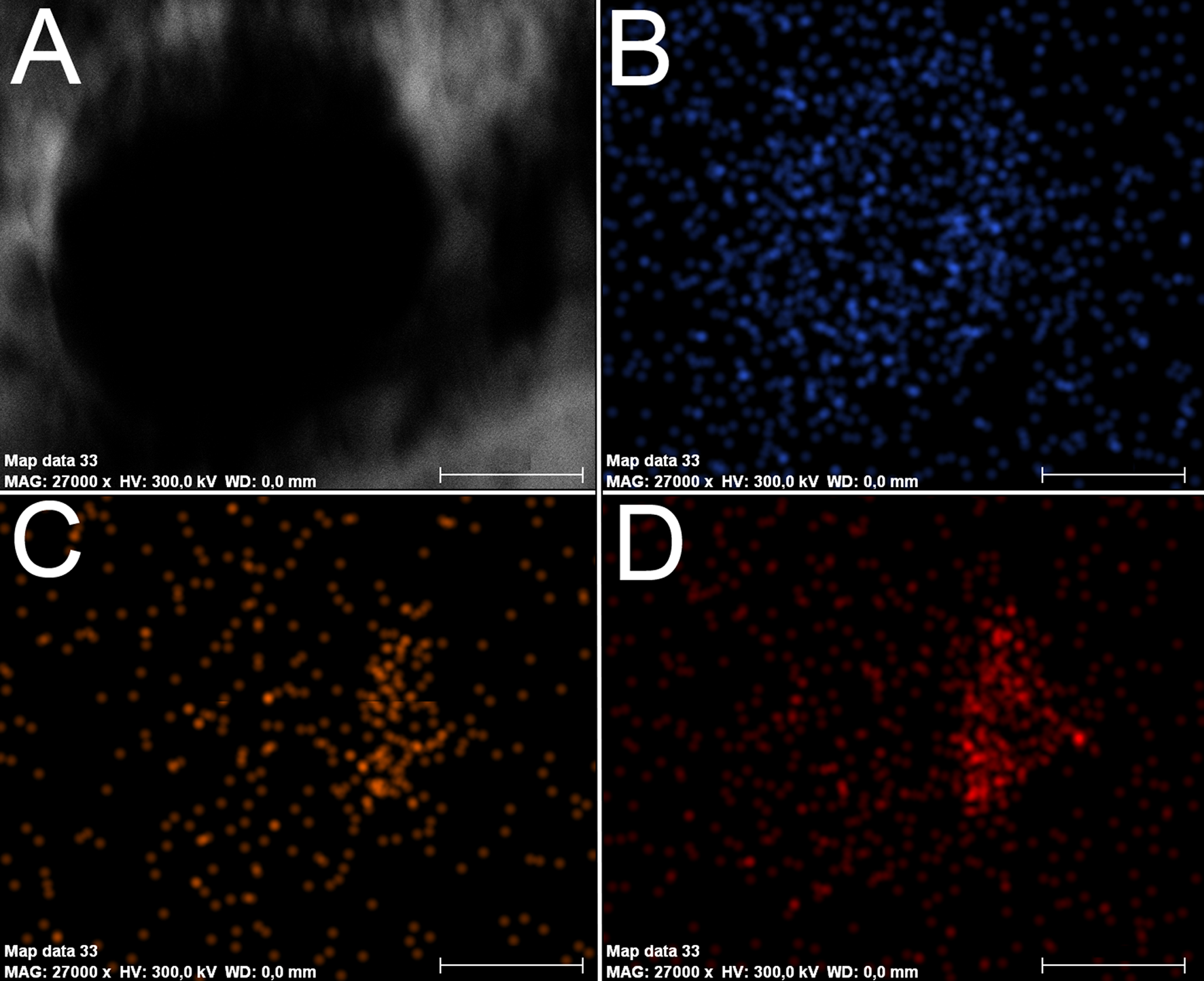

Supplement: Supplementary file 13 [file Image5.TIF]

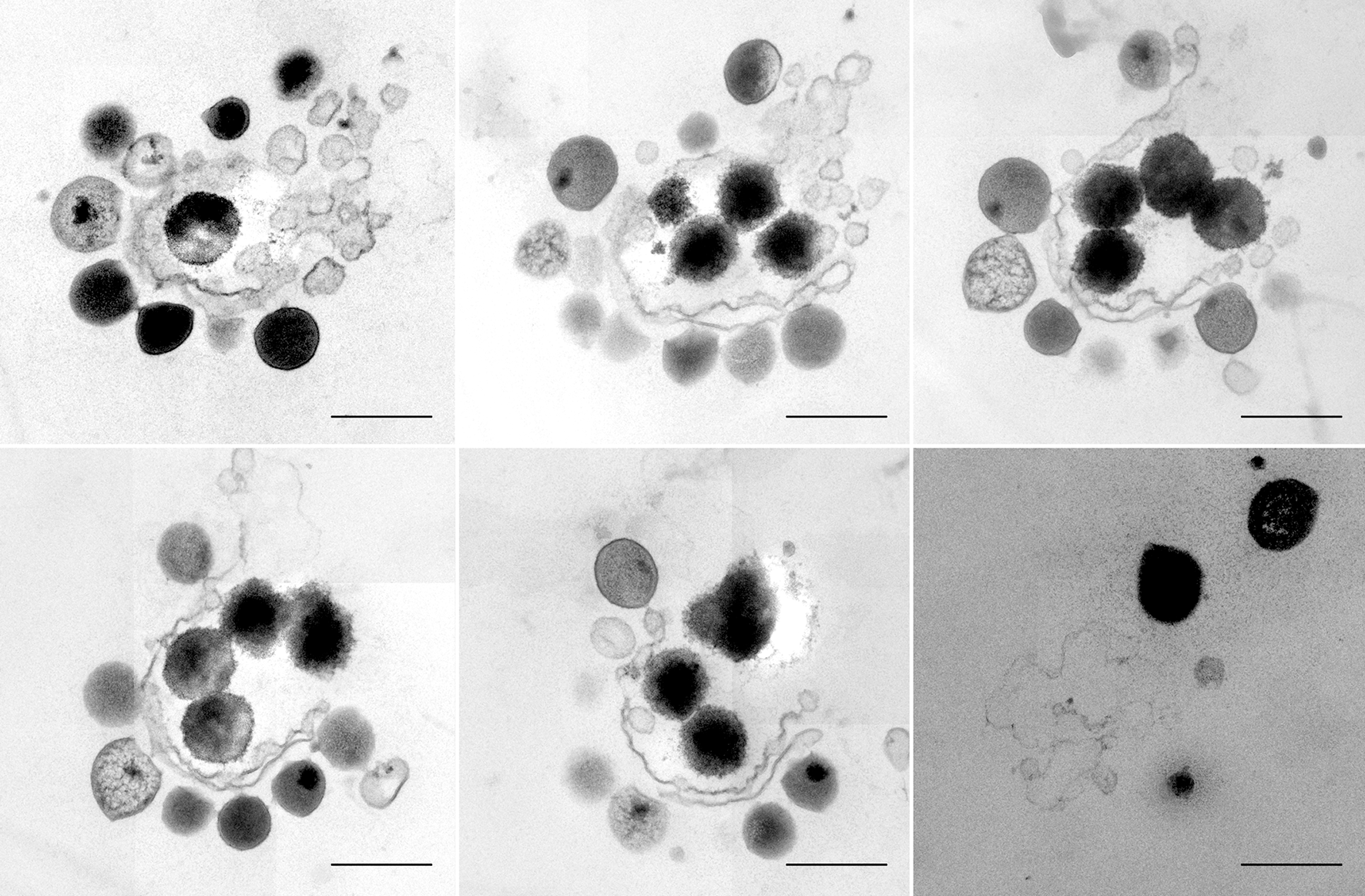

Supplement: Supplementary file 14 [file Image6.TIF]
